# Influence of Temporal and Frequency Selective Patterns Combined with CSP Layers on Performance in Exoskeleton-Assisted Motor Imagery Tasks

**DOI:** 10.3390/neurosci5020012

**Published:** 2024-05-11

**Authors:** Cristian David Guerrero-Mendez, Cristian Felipe Blanco-Diaz, Hamilton Rivera-Flor, Pedro Henrique Fabriz-Ulhoa, Eduardo Antonio Fragoso-Dias, Rafhael Milanezi de Andrade, Denis Delisle-Rodriguez, Teodiano Freire Bastos-Filho

**Affiliations:** 1 Postgraduate Program in Electrical Engineering, Federal University of Espírito Santo (UFES), Vitoria 29075-910, Brazil; cblanco88@uan.edu.co (C.F.B.-D.); hamriver@gmail.com (H.R.-F.); pedro.ulhoa@edu.ufes.br (P.H.F.-U.); teodiano.bastos@ufes.br (T.F.B.-F.); 2 Graduate Program in Mechanical Engineering, Federal University of Espírito Santo (UFES), Vitoria 29075-910, Brazil; eduardo.a.dias@edu.ufes.br (E.A.F.-D.); rafhael.andrade@ufes.br (R.M.d.A.); 3 Postgraduate Program in Neuroengineering, Santos Dumont Institute, Macaiba 59280-000, Brazil; denis.rodriguez@isd.org.br

**Keywords:** passive movements, spatial–spectral filter, brain–computer interfaces, upper limb, EEG

## Abstract

Common Spatial Pattern (CSP) has been recognized as a standard and powerful method for the identification of Electroencephalography (EEG)-based Motor Imagery (MI) tasks when implementing brain–computer interface (BCI) systems towards the motor rehabilitation of lost movements. The combination of BCI systems with robotic systems, such as upper limb exoskeletons, has proven to be a reliable tool for neuromotor rehabilitation. Therefore, in this study, the effects of temporal and frequency segmentation combined with layer increase for spatial filtering were evaluated, using three variations of the CSP method for the identification of passive movement vs. MI+passive movement. The passive movements were generated using a left upper-limb exoskeleton to assist flexion/extension tasks at two speeds (high—85 rpm and low—30 rpm). Ten healthy subjects were evaluated in two recording sessions using Linear Discriminant Analysis (LDA) as a classifier, and accuracy (ACC) and False Positive Rate (FPR) as metrics. The results allow concluding that the use of temporal, frequency or spatial selective information does not significantly (p< 0.05) improve task identification performance. Furthermore, dynamic temporal segmentation strategies may perform better than static segmentation tasks. The findings of this study are a starting point for the exploration of complex MI tasks and their application to neurorehabilitation, as well as the study of brain effects during exoskeleton-assisted MI tasks.

## 1. Introduction

Motor imagery (MI) is a cognitive process that consists in imagining the execution of a movement without presenting a voluntary muscle contraction [1,2,3,4]. The physiological basis of MI is based on the relationship between the electrical activities of the human brain and the actions or states associated with being human [2]. To date, two types of MI have mainly been discussed: kinesthetic (KMI) and visual (VMI). KMI involves the ability to imagine movements by feeling an impression of muscle contractions and sensation of the movement [1]. KMI involves a sensory experience of movements, whereas VMI involves the ability to visualize and imagine movements without the need for sensations in the body. Both types of MI have been involved in intervention for the rehabilitation of people with severe motor limitations using BCIs [5].

Neurological diseases or events such as stroke and Spinal Cord Injury (SCI) are conditions that can generate limitations in mobility and motor functions [6,7]. This is because the communication pathways between the brain and the peripheral nervous system are usually affected, interrupting the synaptic connections that allow the generation of movement or sensory response [8]. Robotic devices emerge as an alternative for the rehabilitation or assistance of these people because mechatronic structures, such as exoskeletons, can support the subject during the performance of tasks, improving independence and quality of life [1,6]. Additionally, these devices, with the repeated and passive execution of movements, can help in the neural retraining of patients with neuromotor impairments [9]. For instance, Pilla et al. [9] designed an upper limb exoskeleton to perform elbow flexion/extension during a continuous protocol in different sessions for the treatment of spasticity in disabled people. Here, the device not only targets therapeutic interventions but is also equipped to carry out biomechanical monitoring of the individual parameters receiving this treatment, highlighting the capabilities of arm exoskeletons for rehabilitation.

Recently, studies reported that MI training may modulate the effects on neuroplasticity and spinal pre-synaptic inhibition, which could benefit people with neuromotor impairments. Moreover, MI-based BCIs have been widely implemented in the motor rehabilitation of upper limb movements, which widely use computational methods in the discrimination of MI tasks from EEG signals. For instance, Padfield et al. in their review discussed the current advantages of EEG-based BCIs to replace functions of the Central Nervous System (CNS), using end-effectors such as robotic devices, prostheses, or virtual reality (VR) therapeutic systems [1]. In this context, active exoskeletons have been the most explored device with MI-based BCI applications because these frameworks can detect a motor intention of the subject and convert this task into a control command, allowing for an assisted movement [1]. For example, different exoskeletons have been used to control finger extension/flexion after detecting upper limb MI from EEG [10,11]. Alternative systems have served as end-effectors to produce movements of flexion/extension in the upper limbs [12]. In contrast, upper joint movements such as the shoulder, elbow, and wrist have also been addressed using EEG signals [13,14,15]. However, challenges in the recognition of mental tasks have been reported because physiological and/or non-physiological artifacts may alter EEG signals [16,17]. Therefore, the implementation of effective computational decoders is still a matter of discussion.

Common Spatial Pattern (CSP) is a powerful technique for MI task discrimination that was first introduced in 1990 to extract special features from EEG signals [18]. CSP aims to find a linear combination of signals so that the variance of one class is maximized while the variance of the other class is minimized [19,20]. Usually, in CSP-based BCIs, after finding spatial patterns highly correlated to a condition, these features are used to train Machine Learning (ML)-based methods to classify MI patterns. For instance, Triana et al. [21] implemented a real-time MI-based BCI system for the identification of sitting and standing tasks using CSP in the feature extraction stage. Rithwik et al. [22] proposed a CSP-based method to decode left and right bidirectional hand movements, highlighting that MI signal processing-based approaches have great potential for incorporation into motor rehabilitation sessions. Cantillo-Negrete et al. designed a CSP-based BCI system to (de)activate a robotic hand orthosis, which was used in the intervention of patients with chronic stroke and healthy subjects [23].

Despite the versatility and effectiveness of the CSP, different variations of this method have been reported in the literature to improve the performance in the identification of MI tasks [24]. For example, Yu et al. [25] implemented a variation called deep CSP to improve MI classification, in which several layers of CSP are implemented to obtain more selective and objective features of MI signals. Ang et al. [19,24] proposed the Filter Bank CSP (FBCSP) configuration to discriminate MI tasks by segmenting the signal into multiple filters before the application of the CSP layer, using a selection criterion based on mutual information. Blanco-Diaz et al. performed a comparative study of spectral and temporal combinations using CSP and FBCSP in the feature extraction stage in two public databases [26]. Finally, variations of CSP, such as unsupervised extraction [27], wavelet CSP [28], or regularized CSP [29], have also been presented with promising outcomes. Although CSP has been reported as a gold standard technique for classifying upper limb MI tasks [13], its default setting is binary classification, which can limit the degrees of neuromotor classification [30,31].

Other efforts have focused on improving the accuracy of computational methods to recognize mental tasks. For instance, Guerrero et al. [32] performed a comparative study between methods based on CSP and Deep Learning (DL) to discriminate hand MI tasks, evaluating performance with different time windows and filter banks. DL achieved better performance than CSP but with additional computational cost. Similarly, Alazrai et al. implemented a DL framework to classify MI tasks on the same hand, achieving interesting results compared to standard approaches [33]. Dong et al. [31] implemented CSP in a configuration of one vs. one to classify multiple MI tasks, such as movement of the right and left upper limbs, foot, and tongue. However, approaches to multiple tasks during flexion/extension arm MI have been little explored.

The use of robotic devices during MI has become an emerging tool for stimulating sensory afferents, improving mechanisms in the cortical control of sensory function [34]. This is associated with two advantages for neurological restoration in people with neuromotor impairments. The first is that the use of haptic–kinesthetic feedback during MI protocols can improve the performance of BCIs task identification algorithms [34,35]. The second is that the use of sensory feedback during MI can re-activate pathways associated with the mechanoreceptors of limbs that have been affected after neurological trauma. This is associated with neural biomarkers [34], which have gained recognition because this information may be used as neurofeedback that can play an important role in induction of neuroplasticity and improvement of proprioception [36,37]. The exploration of cortical behaviors has been presented using hand orthosis [36], arm exoskeletons [34], and robotic gloves [35,37]. However, these works have been focused on binary conditions, such as rest and motion classification, whose limitations are explained above.

Considering that CSP is based on the spatial filtering of EEG signals, the application of several layers combined with spectral or temporal selection could be counterproductive due to loss of information. Furthermore, the authors hypothesized that cortical rhythms may have a recognizable pattern in EEG signals during different states for a similar task, for example, using robotic devices at different speeds during KMI. Therefore, in this study, the performance of three CSP-based methods was evaluated to identify the MI of upper limb extension/flexion during passive movement from an exoskeleton at two speeds. Specifically, EEG patterns related to passive movement vs. MI+passive movement in flexion/extension tasks of the left upper limb were identified. It is worth mentioning that it is still relevant in the literature to study the effects related to the application of specific methods, such as CSP. This pattern classification could facilitate understanding of brain behavior during different tasks with the same limb, which may lead to the development of more personalized neurorehabilitation systems.

## 2. Materials and Methods

### 2.1. CSP-Based Methods

**Figure 1:**
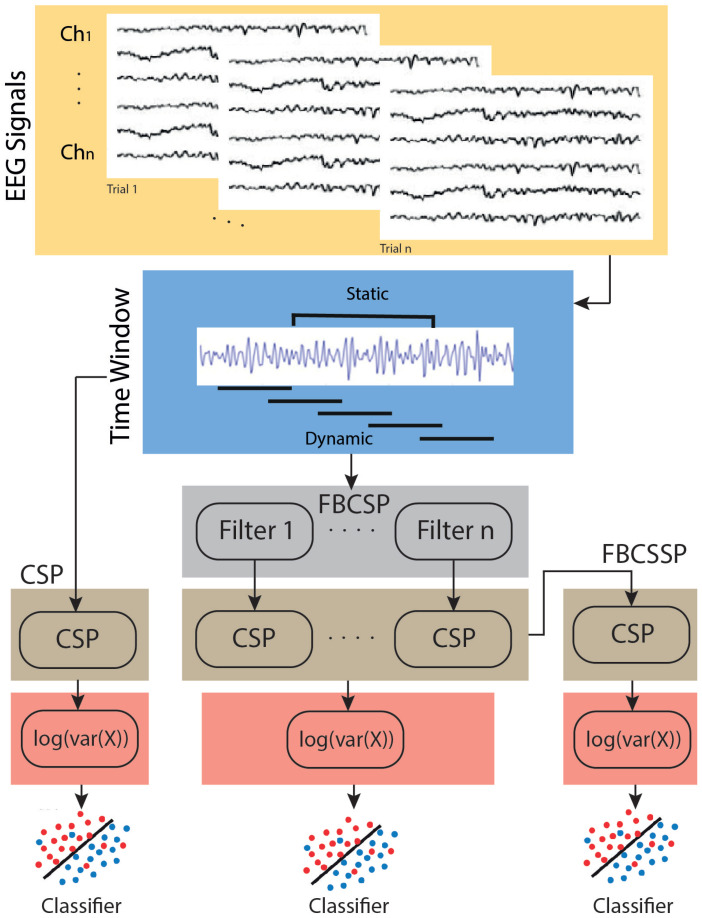
Block diagram of the CSP-based methods (CSP, FBCSP, and FBCSSP) implemented in this study.

This section presents the CSP configurations used in this study: CSP, FBCSP, and Filter Bank Common Spectral Spatial Patterns (FBCSSP). For more information on each of the methods, please refer to our previous works [26,32]. A graphical representation of each of the CSP variations is shown in Figure 1.

#### 2.1.1. Classic CSP

CSP is classified as a supervised technique used to identify between different classes (usually two classes). Thus, a class label was required to identify the weight matrices during pattern generation [25]. The principle of this technique is the implementation of a spatial filter, whose main function can be elucidated through the subsequent equation:

(1) $$\tilde{Y}=W^{T}Y,$$

where the matrix $W\in R^{p\times C}$ encompasses the weights allocated for spatial filtering, whereas $\tilde{Y}\in R^{t\times P}$ denotes the resultant EEG data post-application of the weighted matrix. The symbol $Y\in R^{t\times C}$ denotes the filtered EEG signal, *t* represents the number of samples over time, and *C* is the EEG channels used. Note that *P* corresponds to the number of spatial patterns involved.

On the other hand, the calculation of the matrix *W* is performed by $W=P^{T}A$, where *P* is a relationship between a set of eigenvectors and a diagonal matrix of eigenvalues, and *A* is a set of associated eigenvectors [25]. Finally, the features extracted using the CSP method correspond to the logarithm of variance from the matrix $\tilde{Y}$, which are subsequently used to identify MI patterns, usually in binary classification. In this study, three patterns were used to calculate the weight matrix, obtaining six CSPs (one per class).

#### 2.1.2. Selective Frequency CSP

Spectral feature segmentation was performed using a filter bank prior to application of the CSP layer (FBCSP). EEG signals were segmented into several frequency bands of interest, mainly around the Mu (μ, 8–12 Hz) and Beta (β, 14–30 Hz) bands, and subsequently a CSP layer was applied in each of the frequency sub-bands. Several authors have reported the use of a mutual information-based feature selection stage, which was implemented in this study to obtain more selective features from EEG signals [19]. In addition, previous works have demonstrated that filter banks of 8–15 Hz, 15–22 Hz, and 22–30 Hz show adequate accuracy rates for binary classification [26,32]. Note that this method uses spectral filtering (using the filter bank) and spatial filtering through the CSP layer, which is implemented following the same configuration presented in the previous section.

#### 2.1.3. Combined CSP Layers with Selective Frequency

The variation implemented to increase the number of CSP layers uses a filter bank, previously extracted using FBCSP, and applies an additional CSP layer that combines each of the sub-CSPs calculated for each filter bank [32,38]. This method is called FBCSSP, see Figure 1. Note that in this variation, two CSP layers are implemented using the same configurations as presented in the previous sections. A description of each layer is presented below.

- Layer 1: This layer enables the extraction of the CSP from filtered signals after employing the filter bank. Its importance stems from its capability to emphasize differences in variances between classes for particular frequency-selective patterns [38].
- Layer 2: CSP is used to generate more selective features associated with the spatial and spectral domains. These parameters are computed by linearly projecting the weights with the output of each CSP in layer 1.

### 2.2. Experimental Protocol

#### 2.2.1. Participant

Ten healthy individuals participated in this study (six male and four female), ranging in age from 21 to 48 years (29 ± 9), and without any neurological, psychological or other health conditions reported. The statistical power of the sample size was estimated using G*power software to suggest whether the results of the performance metrics can be compared with the literature [39,40]. Wilcoxon signed rank was used with a value of α=0.05, an effect size of d=2.13, and a sample size of n=10. The results of this test yielded a statistical power of 0.9, which is within the ranges reported in previous studies [40].

Ethics Committee: All volunteers provided their consent voluntarily and confirmed their agreement through an informed consent process, in accordance with the principles outlined in the Declaration of Helsinki. The research protocol was approved by the Ethics Committee of the Federal University of Espírito Santo (UFES) and registered under the protocol number CAAE:39410614.6.0000.5060.

#### 2.2.2. EEG Collection

**Figure 2:**
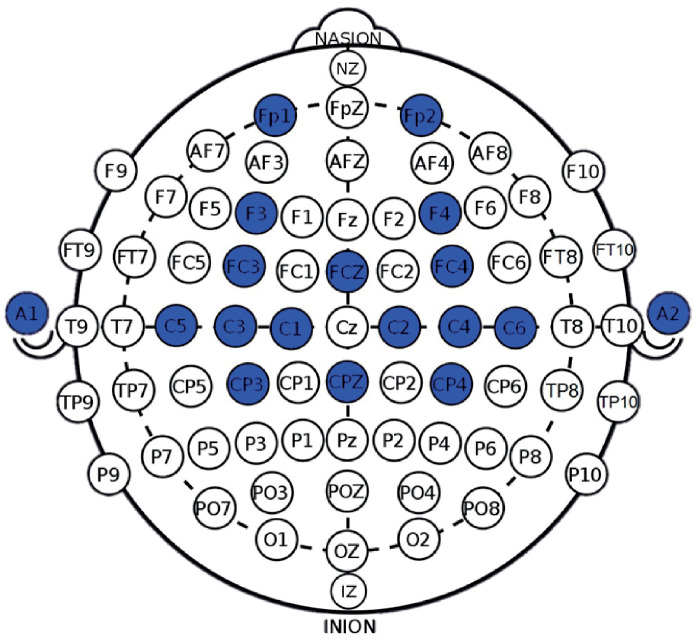
EEG locations used in this study considering 10–20 international system.

The EEG data were collected using a total of 16 electrodes placed on the scalps of participants according to the 10–20 international system. The recorded EEG channels were FP1, FP2, F3, F4, FC3, FCz, FC4, C5, C3, C1, C2, C4, C6, CP3, CPz and CP4, sampled at a rate of 125 Hz, with references A1 and A2 placed in the earlobes as shown in Figure 2. To acquire EEG signals and monitor impedance, the OpenBCI Cyton + Daisy device was used along with its Graphic User Interface (GUI), ensuring that the impedance of the channels with the scalp remained below 20 kΩ. The selection of these specific channels was established by prior research, which emphasized their relevance within sensorimotor regions to discriminate tasks related to MI of upper limb movements [26].

#### 2.2.3. Procedures

**Figure 3:**
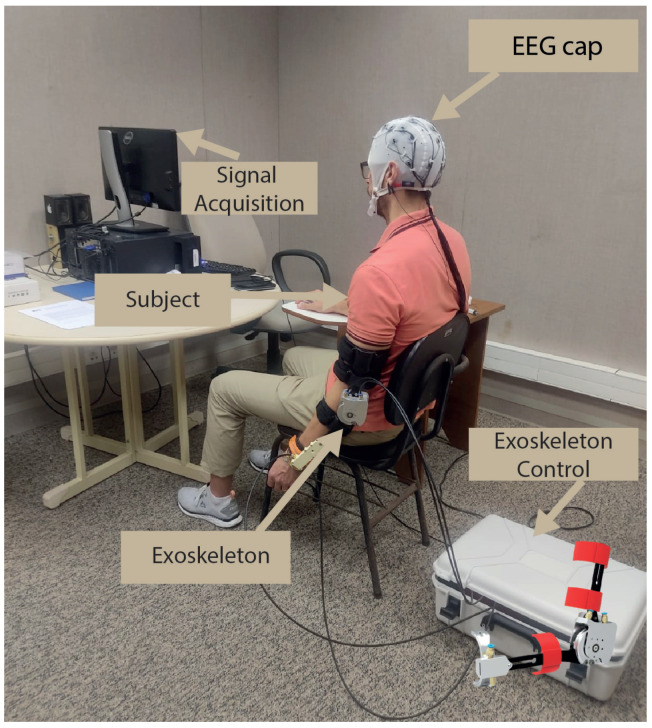
Experimental setup for data collection using the robotic exoskeleton.

During procedures, MI is related to flexion/extension movements of the upper limbs (specifically, the arm and elbow) while using an exoskeleton. More information about the exoskeleton can be found in [41,42]. Figure 3 illustrates the setup of the acquisition environment and the instrumented exoskeleton on the subjects. For data recording, the protocol, comprising the following procedural steps, was executed in sequence as follows:

- Passive movement (baseline): The exoskeleton initiates passive movements at a minimal speed, set at 30 rotations per minute (rpm). During this phase, lasting 120 s, the subject performs passive flexion/extension tasks facilitated by the exoskeleton.
- Beep: A beep is used to signal the beginning of each trial, lasting 1.25 s. Its purpose is to inform the subject about the beginning of a new repetition cycle.
- No-action: During the no-action period, which spans from 2 to 3 s, the subject remains in a state of relaxation and rest, with no specific task or action required.
- MI+passive movement: MI plus passive movement are incorporated, with variations in two speeds—30 and 85 rpm over a duration of 10 s. In this phase, the subject is instructed to mentally simulate (MI) the performance of flexion/extension movements while simultaneously receiving passive movements from the exoskeleton.

**Figure 4:**
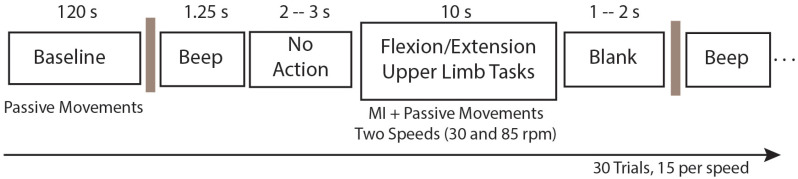
Protocol sequence followed by the subjects for task collection (passive movement and MI+passive movement).

The recordings were conducted in a noise-isolated environment, with only the subject and a researcher present. Note that visual cues were not provided through a screen. Instead, instructions were transmitted through both auditory cues (beep) and the sensation of movement generated by the exoskeleton. The protocol for each subject included 30 trials, spanning from the initial beep to the MI+passive movement phase. Within these trials, 15 were performed at a minimum speed (30 rpm), followed by subsequent 15 repetitions at a higher speed (85 rpm), resulting in a total of 30 trials. The speed variation between trials was randomized to avoid predictability. Each subject underwent the protocol twice, constituting two sessions with a 5-min interval between them. The sequential order of the protocol is shown in Figure 4.

### 2.3. Signal Pre-Processing and Evaluation

#### 2.3.1. EEG Signal Pre-Processing

EEG signals were filtered using a 5th order zero-phase Butterworth filter in the frequency band from 8 to 30 Hz to obtain spectral information in the Mu (μ) and Beta (β) bands. Subsequently, the signals were segmented temporally, implementing two strategies, one continuous (dynamic) and one static. The continuous one (TW0) corresponded to segmenting the signals in time windows of 1 s overlapped at 50%, while the static one (TW3) corresponded to selecting a specific time range, in this case, from 1 to 6 s. This allows to evaluate the temporal behavior in the identification of MI patterns.

Note that spatial filters, such as Common Average Reference (CAR), were not implemented in the preprocessing stage in order to avoid combining undesirable spatial filters. After filtering and segmenting the signals, the CSP methods described above were implemented.

#### 2.3.2. Evaluation

To classify the features associated to each task using the CSP methods, Linear Discriminant Analysis (LDA) was implemented. This classifier was used due to its low computational cost and the fact that LDA has been classified as a standard ML method to identify MI patterns [43]. As aforementioned, the objective of this study is to identify passive movement tasks (baseline) vs. MI+passive movement at each of the exoskeleton speeds: low (30 rpm) and high (85 rpm).

The k-fold cross-trial validation was implemented as a validation method using five folds, segmenting the data into 80% trials for training and 20% for testing. The performance metrics used were accuracy (*ACC*) and False Positive Rate (*FPR*); see Equation (2). These metrics were calculated using the confusion matrix for binary discrimination, where TP corresponds to true positives, TN to true negatives, FP to false positives, and FN to false negatives:

(2) $$\begin{array}{c} ACC=\frac{TP+TN}{TP+FN+FP+TN}\\ FPR=\frac{FP}{FP+TN} \end{array}$$

Additionally, a statistical significance analysis was performed to determine the effects on performance in the use of temporal and frequency segmentation together with the application of CSP layers. For this, the performance data of all subjects were evaluated using Shapiro–Wilk and Levene tests to observe the type of distribution and homogeneity of variances. Considering that the data presented a high probability of belonging to a normal distribution with homogeneous variances, the two-sample *t*-test with a significance threshold of 0.05 was applied. The alternative hypothesis is that the CSP method presented better performance metrics in ACC and FPR than the FBCSP and FBCSSP variants. Finally, the null hypothesis is the opposite.

## 3. Results

**Figure 5:**
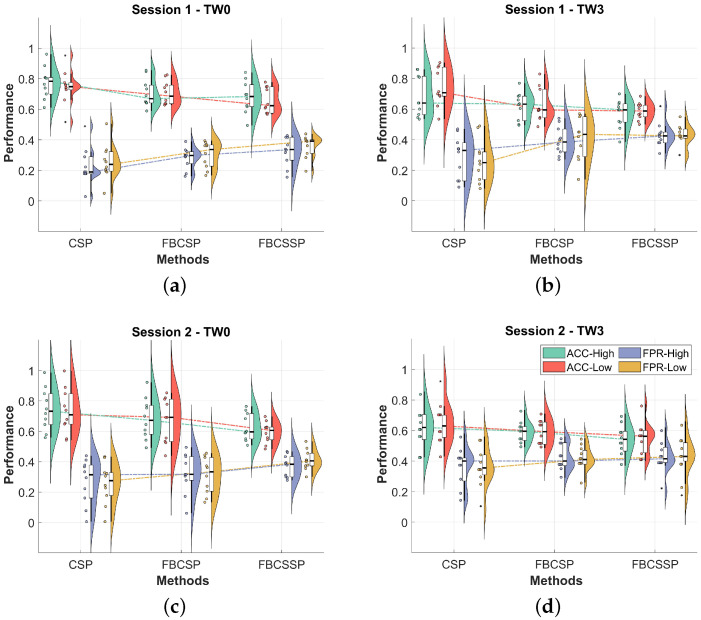
Performance in the identification of passive movement vs. MI+passive movement in the three CSP variations to determine the effects of temporal and frequency segmentation, and the application of CSP layers. (**a**) Session 1—TW0; (**b**) Session 1—TW3; (**c**) Session 2—TW0; (**d**) Session 2—TW3.

The performance in the identification of passive movement vs. MI+passive movement in the left upper limb flexion/extension tasks using the robotic exoskeleton at two different speeds is presented in Figure 5. The results are shown in a joint representation using a boxplot and semi-violinplot, which allow observing the distribution and density of the data to obtain the variability and concentration in certain areas of the distribution region. The results are presented for the three CSP variations implemented (classical CSP, FBCSP, and FBCSSP), for the two types of temporal segmentation, and for the two recording sessions. The results for each speed are shown for each metric, where ACC high (high speed) is shown in green, ACC low (low speed) in red, FPR high in purple, and FPR low in yellow, respectively.

The behavior of the results reveals that the use of temporal and frequency segmentation, as well as the application of CSP layers, degrades the performance in the identification of passive movement vs. MI+passive movement. This behavior is more noticeable in the static temporal segmentation represented by TW3. Regarding the distribution of the metrics data, it is possible to observe that in most of them, there is a homogeneous density.

In order to evaluate whether the effects on performance are significant, i.e., whether frequency segmentation together with the application of CSP layers degrades the performance in task identification, the differences (significant or non-significant) in the performance comparison between the methods are presented in Table 1 and Table 2. From these, it is possible to observe that there are significant differences between the CSP and FBCSSP methods for TW0 and TW3 window segmentation. Furthermore, for TW3, the CSP and FBCSP methods also presented significant differences. In contrast, for session 2, CSP and FBCSSP presented significant differences in performance; however, these differences were only presented for the temporal segmentation TW0. For the other comparisons between methods, the differences were not significant. These effects were for the two performance metrics, ACC and FPR. These results allow determining that the FBCSSP method presents significantly lower performance than the CSP for the identification of passive movement vs. MI+passive movement.

## 4. Discussion

This paper presents the effects of performance on passive movement vs. MI+passive movement identification, employing three variations of the CSP method based on temporal and frequency segmentation, and CSP layer augmentation. Passive movements were generated using an upper limb exoskeleton that performs left arm flexion/extension tasks at two different speeds. The results of this study allow determining that the use of selective information in time, frequency, or space does not significantly improve the performance in the identification tasks evaluated using CSP variations. This result is in agreement with the literature. For instance, Ùbeda et al. implemented a linear decoder to estimate hand movements during passive motion, where low-performance metrics were found, suggesting that further exploration related to the frequency band is required [44].

In our previous studies related to temporal and frequency evaluation and CSP layer enhancement, we determined time windows and specific filter banks to find EEG patterns that allow to differentiate MI tasks with greater accuracy [26,32,38]. For instance, in [26], these methods were compared to decode MI of the right and left hands in two databases, finding significant differences when varying the length of the time window and the filter bank configurations. In [32], CSP-based methods were compared with artificial neural networks, where we found accuracy improvements using these DL techniques but with a higher computational cost. It should be noted that in these previous studies were based on left- and right-hand classification, which diverge from those presented during this study. In another approach reported in [38], CSP techniques were evaluated during the execution of lower limb movements, such as classification between pedaling and non-pedaling tasks from EEG signals.

To the best of our knowledge, this is the first time that an analysis with EEG signals during KMI and an arm exoskeleton at different speeds that performs flexion/extension tasks is implemented. However, previous studies have reported findings during similar experiments. For instance, Barios et al. performed an analysis of cortical rhythms during the use of a BCI with haptic, visual, and auditory feedback [34]. Haptic feedback was delivered by an arm exoskeleton, where the results suggest that phase synchronization of cortical rhythms can identify the identification of temporal edges in MI. However, the passive movement was configured with constant velocity and time execution of 1–2 s. Gonzalez-Cely et al. performed a preliminary study that demonstrated that the passive motion of a robotic glove may improve the performance of ML techniques to classify open/close-hand MI tasks [35]. This proposal also used constant velocity during the protocol, which may limit the scope. Guggenberg et al. implemented a strategy to classify KMI from EEG signals in a protocol that employed a robotic hand orthosis and a functional electrical stimulator [36]. This approach reached an accuracy rate above 0.6 using information from the Beta (β) band. Ono et al. [37] experimented with neurofeedback training, using a robotic hand and action observation during several sessions on different days. It was observed that events related to desynchronization increased after training, suggesting that the use of robotic devices and proprioception stimulation may improve the behavior of cortical rhythms. However, the experiment was performed with constant movement visualization. Cantillo-Negrete et al. [23] implemented a BCI based on a hand orthosis that was commanded by MI detection with CSP, allowing for activation and deactivation (binary classification). Improvements in accuracy were reached after different sessions by post-stroke patients, which highlights the potential of these systems for clinical trials.

Other works have demonstrated the behavior of cortical rhythms during the measurement of EEG signals and the use of upper limb robotic devices. For instance, the systems reported in [10,11] suggested the feasibility of BCIs to control finger movements in neurorehabilitation strategies. Moreover, other robotics-based BCIs were designed with the objective of rehabilitating spasticity over joints, such as the wrist, elbow, or shoulder [14,15]. The tasks evaluated in the aforementioned works are based on left- and right-hand MI classification, rest and MI classification, or movement and rest classification from EEG signals, i.e., binary classification of states. Therefore, other efforts have concentrated on the classification of multiple tasks. For example, Dong et al., implemented CSP-based methods in a configuration of one vs. one to classify left- and right-hand, tongue, and foot MI [31]. Nevertheless, they involved different tasks on the limb, which would make classifying the EEG signal easier. Meanwhile, Alazrai et al. used neural networks to classify MI tasks from the same limb, highlighting that this approach may be more challenging due to cortical behaviors [33]. However, this approach does not employ passive movements from a robotic exoskeleton.

In this context, we hypothesized that the use of a robotic arm exoskeleton at different speeds could enhance cortical rhythms which could facilitate the classification of upper limb KMI tasks. An adequate classification score and False Positive Rate were found, considering the literature reports [31,32,33,36]. Considering the working principle of CSP-based methods [19], our results suggest that information in the time domain cannot be fully discriminatory when the KMI is under different conditions, which is a limitation for spatial filtering methods. Further analysis in the frequency domain may complement or contradict this statement in the future.

On the other hand, it seems that the CSP method without the use of a filter bank had better performance metrics compared to FBCSP and FBCSSP. This reported effect may be due to several factors. The most supported one in the literature is that passive assistance during EEG protocols is still a matter of discussion in the neuroscience field, as some authors report adequate performance metrics using slow cortical rhythms (delta band < 4 Hz) for detection [44], while others mention only the Beta band in their BCIs [36]. For this reason, the selection of an appropriate filter bank is not trivial. In previous studies [26,32,38], our findings with FBCSSP were better than those with conventional CSP using the 3-filter configuration of 8–15 Hz, 15–22 Hz, and 22–30 Hz, which was our motivation. However, it was possible to observe that further analysis with different configurations is necessary to improve the performance metrics of the classifiers.

In addition, it was superficially observed that the effects (positive or negative) on performance depend on the type of tasks to be identified and the type of protocol implemented. For example, this type of effect also depends on the recording equipment, the impedance and type of electrodes, the emotional conditions of the subjects, and the methods evaluated, among other considerations [17,45]. Furthermore, the type of ML method used for task recognition may influence the classification tasks. Thus, it is recommended to explore and compare the performance along with more complex methods such as Support Vector Machine (SVM) or Extreme Learning Machine (ELM). Furthermore, another main factor may be due to the type of task evaluated, which may be associated with a complete task that requires more concentration and experience to generate discriminative patterns [46]. Therefore, being a complex task, temporal and frequency segmentation may degrade associated patterns and decrease performance. Also, the application of double spatial filtering (CPS layers), which selects better distributed spatial patterns, may be counterproductive for the recognition and filtering of task-relevant information for discrimination. Finally, another cause of low performance in the FBCSP and FBCSSP methods is due to the implementation of feature selection based on mutual information, which, in principle, would select more specific patterns; however, this may cause a negative effect considering the loss of information when selecting features.

Interventions with repetitive flexion/extension movements of the upper limbs using exoskeletons have been proposed not only for the treatment of joint spasticity but also as a tool for monitoring information related to the evolution of patients with neurological impairments [9]. In this context, our results allowed determining a surface difference between low and high speeds generated by the passive movement of the exoskeleton, which has not been previously reported. This is a starting point to further explore the effect of kinesthetic velocities on cortical rhythms during the execution of MI tasks. We consider that our method can be extended to the design of more robust BCIs that allow for more personalized rehabilitation, i.e., a human–robot interface that allows repetitive cycling at different speeds using intention by MI. This is not only for neurorehabilitation purposes but can also be used as a methodology to follow the neural evolution of patients [9]. However, instrumental monitoring with EEG signals requires further evidence.

Eventually, the exploration of cortical rhythms is still under study under different conditions. Our results allowed determining differences in classifier performance during passive movement tasks. Different performance metrics were found with respect to each condition, suggesting that the cortical patterns between speeds may differ, although minimally. For this reason, different and more complex approaches are suggested. The CSP-based techniques were able to discriminate the different tasks with an accuracy rate above chance, showing that our technique is feasible for binary classification and can be extended in a multiclass version. These results mark a starting point for explorations of upper limb EEG signals using robotic assistance under different conditions, which has the potential to be implemented in the construction of neurofeedback biomarkers or sensory feedback in BCIs for neurorehabilitation.

Finally, this study had some limitations. On the one hand, the sample size used in this study could limit the general effects of the methods’ performance, limiting also the comparison of other physiological factors, such as sex, age, and mental conditions. On the other hand, other ML-based methods, which could present a more robust classification performance in temporal, frequency, and layered CSP segmentation, were not employed in this study.

## 5. Conclusions

This study concludes that the use of temporal and frequency selective segmentation, together with a layer increase for spatial filtering evaluated in three variations of the CSP method (classical CSP, FBCSP, and FBCSSP), does not significantly improve performance in the identification of passive movement vs. MI+passive movement in assisting left arm flexion/extension tasks by using a left upper limb exoskeleton. However, differences between classifier performance during KMI at different speeds of the robotic device were found, suggesting that the cortical patterns between speeds may be different but in a very minimal way. It was also verified that dynamic temporal segmentation (considering the whole signal time) presented higher robustness compared to static temporal segmentation (in a given period of time).

Future studies will focus on evaluating these types of complex tasks in different temporal, frequency, and spatial-filtering strategies, as well as recognition methods to find more discriminative patterns to direct research towards BCIs for neuromotor rehabilitation based on complex tasks.

## Figures and Tables

**Table 1:** Significant differences between CSP variations for Session 1.

	TW0				TW3			
	**ACC-H**	**ACC-L**	**FPR-H**	**FPR-L**	**ACC-H**	**ACC-L**	**FPR-H**	**FPR-L**
CSP vs. FBCSP	∼	∼	∼	∼	∼	†	†	†
CSP vs. FBCSSP	†	†	†	†	†	†	†	†
FBCSP vs. FBCSSP	∼	∼	∼	∼	∼	∼	∼	∼

∼ means no significant differences; † means significant differences (*p* < 0.05).

**Table 2:** Significant differences between CSP variations for Session 2.

	TW0				TW3			
	**ACC-H**	**ACC-L**	**FPR-H**	**FPR-L**	**ACC-H**	**ACC-L**	**FPR-H**	**FPR-L**
CSP vs. FBCSP	∼	∼	∼	∼	∼	∼	∼	∼
CSP vs. FBCSSP	†	†	†	†	∼	∼	∼	∼
FBCSP vs. FBCSSP	∼	∼	∼	†	∼	∼	∼	∼

∼ means no significant differences; † means significant differences (*p* < 0.05).

## Data Availability

Data are currently unavailable.

## References

[1] Padfield N., Zabalza J., Zhao H., Masero V., Ren J. (2019). EEG-Based Brain-Computer Interfaces Using Motor-Imagery: Techniques and Challenges. Sensors.

[2] Herranz-Gómez A., Gaudiosi C., Angulo-Díaz-Parreño S., Suso-Martí L., La Touche R., Cuenca-Martínez F. (2020). Effectiveness of motor imagery and action observation on functional variables: An umbrella and mapping review with meta-meta-analysis. Neurosci. Biobehav. Rev..

[3] Pfurtscheller G., Lopes da Silva F. (1999). Event-related EEG/MEG synchronization and desynchronization: Basic principles. Clin. Neurophysiol..

[4] Monteiro K.B., dos Santos Cardoso M., da Costa Cabral V.R., Dos Santos A.O.B., da Silva P.S., de Castro J.B.P., de Souza Vale R.G. (2021). Effects of motor imagery as a complementary resource on the rehabilitation of stroke patients: A meta-analysis of randomized trials. J. Stroke Cerebrovasc. Dis..

[5] Chholak P., Niso G., Maksimenko V.A., Kurkin S.A., Frolov N.S., Pitsik E.N., Hramov A.E., Pisarchik A.N. (2019). Visual and kinesthetic modes affect motor imagery classification in untrained subjects. Sci. Rep..

[6] Khan M.A., Das R., Iversen H.K., Puthusserypady S. (2020). Review on motor imagery based BCI systems for upper limb post-stroke neurorehabilitation: From designing to application. Comput. Biol. Med..

[7] Marquez-Chin C., Popovic M.R. (2020). Functional electrical stimulation therapy for restoration of motor function after spinal cord injury and stroke: A review. Biomed. Eng. Online.

[8] Qin C., Yang S., Chu Y.H., Zhang H., Pang X.W., Chen L., Zhou L.Q., Chen M., Tian D.S., Wang W. (2022). Signaling pathways involved in ischemic stroke: Molecular mechanisms and therapeutic interventions. Signal Transduct. Target. Ther..

[9] Pilla A., Trigili E., McKinney Z., Fanciullacci C., Malasoma C., Posteraro F., Crea S., Vitiello N. (2020). Robotic rehabilitation and multimodal instrumented assessment of post-stroke elbow motor functions—A randomized controlled trial protocol. Front. Neurol..

[10] Cantillo-Negrete J., Carino-Escobar R.I., Carrillo-Mora P., Elias-Vinas D., Gutierrez-Martinez J. (2018). Motor imagery-based brain-computer interface coupled to a robotic hand orthosis aimed for neurorehabilitation of stroke patients. J. Healthc. Eng..

[11] Mukherjee P., Roy A.H. (2024). EEG sensor driven assistive device for elbow and finger rehabilitation using deep learning. Expert Syst. Appl..

[12] Xu B., Song A., Zhao G., Xu G., Pan L., Yang R., Li H., Cui J., Zeng H. (2015). Robotic neurorehabilitation system design for stroke patients. Adv. Mech. Eng..

[13] Ang K.K., Chua K.S.G., Phua K.S., Wang C., Chin Z.Y., Kuah C.W.K., Low W., Guan C. (2015). A randomized controlled trial of EEG-based motor imagery brain-computer interface robotic rehabilitation for stroke. Clin. EEG Neurosci..

[14] Vourvopoulos A., Pardo O.M., Lefebvre S., Neureither M., Saldana D., Jahng E., Liew S.L. (2019). Effects of a brain-computer interface with virtual reality (VR) neurofeedback: A pilot study in chronic stroke patients. Front. Hum. Neurosci..

[15] Vourvopoulos A., Marin-Pardo O., Neureither M., Saldana D., Jahng E., Liew S.L. (2019). Multimodal Head-Mounted Virtual-Reality Brain-Computer Interface for Stroke Rehabilitation: A Clinical Case Study with REINVENT. Proceedings of the Virtual, Augmented and Mixed Reality. Multimodal Interaction: 11th International Conference, VAMR 2019, Held as Part of the 21st HCI International Conference, HCII 2019.

[16] Huang Z., Wang M. (2021). A review of electroencephalogram signal processing methods for brain-controlled robots. Cogn. Robot..

[17] Guerrero-Mendez C.D., Blanco-Díaz C.F., Jaramillo-Isaza S., Bastos-Filho T.F., Ruiz-Olaya A.F. (2024). Artificial Intelligence Applied to Neuromotor Rehabilitation Engineering: Advances and Challenges. Computational Approaches in Biomaterials and Biomedical Engineering Applications.

[18] Koles Z.J., Lazar M.S., Zhou S.Z. (1990). Spatial patterns underlying population differences in the background EEG. Brain Topogr..

[19] Ang K.K., Chin Z.Y., Wang C., Guan C., Zhang H. (2012). Filter Bank Common Spatial Pattern Algorithm on BCI Competition IV Datasets 2a and 2b. Front. Neurosci..

[20] Wu W., Chen Z., Gao X., Li Y., Brown E.N., Gao S. (2014). Probabilistic common spatial patterns for multichannel EEG analysis. IEEE Trans. Pattern Anal. Mach. Intell..

[21] Triana-Guzman N., Orjuela-Cañon A.D., Jutinico A.L., Mendoza-Montoya O., Antelis J.M. (2022). Decoding EEG rhythms offline and online during motor imagery for standing and sitting based on a brain-computer interface. Front. Neuroinform..

[22] Rithwik P., Benzy V., Vinod A. (2022). High accuracy decoding of motor imagery directions from EEG-based brain computer interface using filter bank spatially regularised common spatial pattern method. Biomed. Signal Process. Control.

[23] Cantillo-Negrete J., Carino-Escobar R.I., Carrillo-Mora P., Rodriguez-Barragan M.A., Hernandez-Arenas C., Quinzaños-Fresnedo J., Hernandez-Sanchez I.R., Galicia-Alvarado M.A., Miguel-Puga A., Arias-Carrion O. (2021). Brain-computer interface coupled to a robotic hand orthosis for stroke patients’ neurorehabilitation: A crossover feasibility study. Front. Hum. Neurosci..

[24] Ang K.K., Chin Z.Y., Zhang H., Guan C. Filter bank common spatial pattern (FBCSP) in brain-computer interface. Proceedings of the 2008 IEEE International Joint Conference on Neural Networks (IEEE World Congress on Computational Intelligence).

[25] Yu N., Yang R., Huang M. (2022). Deep Common Spatial Pattern based Motor Imagery Classification with Improved Objective Function. Int. J. Netw. Dyn. Intell..

[26] Blanco-Diaz C.F., Antelis J.M., Ruiz-Olaya A.F. (2022). Comparative analysis of spectral and temporal combinations in CSP-based methods for decoding hand motor imagery tasks. J. Neurosci. Methods.

[27] Martin-Clemente R., Olias J., Cruces S., Zarzoso V. (2019). Unsupervised Common Spatial Patterns. IEEE Trans. Neural Syst. Rehabil. Eng..

[28] Zhang S., Zhu Z., Zhang B., Feng B., Yu T., Li Z. (2020). The CSP-Based New Features Plus Non-Convex Log Sparse Feature Selection for Motor Imagery EEG Classification. Sensors.

[29] An Y., Lam H.K., Ling S.H. (2023). Multi-classification for EEG motor imagery signals using data evaluation-based auto-selected regularized FBCSP and convolutional neural network. Neural Comput. Appl..

[30] Padfield N., Camilleri K., Camilleri T., Fabri S., Bugeja M. (2022). A Comprehensive Review of Endogenous EEG-Based BCIs for Dynamic Device Control. Sensors.

[31] Dong E., Li C., Li L., Du S., Belkacem A.N., Chen C. (2017). Classification of multi-class motor imagery with a novel hierarchical SVM algorithm for brain-computer interfaces. Med. Biol. Eng. Comput..

[32] Guerrero-Mendez C.D., Blanco-Diaz C.F., Ruiz-Olaya A.F., López-Delis A., Jaramillo-Isaza S., Milanezi Andrade R., Ferreira De Souza A., Delisle-Rodriguez D., Frizera-Neto A., Bastos-Filho T.F. (2023). EEG motor imagery classification using deep learning approaches in naïve BCI users. Biomed. Phys. Eng. Express.

[33] Alazrai R., Abuhijleh M., Alwanni H., Daoud M.I. (2019). A Deep Learning Framework for Decoding Motor Imagery Tasks of the Same Hand Using EEG Signals. IEEE Access.

[34] Barios J.A., Ezquerro S., Bertomeu-Motos A., Nann M., Badesa F.J., Fernandez E., Soekadar S.R., Garcia-Aracil N. (2019). Synchronization of slow cortical rhythms during motor imagery-based brain–machine interface control. Int. J. Neural Syst..

[35] González-Cely A.X., Blanco-Díaz C.F., Guerrero-Mendez C.D., Bastos-Filho T.F. Hand Motor Imagery Identification Using Machine Learning Approaches in a Protocol Based on Visual Stimuli and Passive Movement. Proceedings of the 2023 IEEE Colombian Caribbean Conference (C3).

[36] Guggenberger R., Heringhaus M., Gharabaghi A. (2020). Brain-machine neurofeedback: Robotics or electrical stimulation?. Front. Bioeng. Biotechnol..

[37] Ono Y., Wada K., Kurata M., Seki N. (2018). Enhancement of motor-imagery ability via combined action observation and motor-imagery training with proprioceptive neurofeedback. Neuropsychologia.

[38] Blanco-Díaz C.F., Guerrero-Mendez C.D., Delisle-Rodriguez D., Jaramillo-Isaza S., Ruiz-Olaya A.F., Frizera-Neto A., Ferreira de Souza A., Bastos-Filho T. (2024). Evaluation of temporal, spatial and spectral filtering in CSP-based methods for decoding pedaling-based motor tasks using EEG signals. Biomed. Phys. Eng. Express.

[39] Faul F., Erdfelder E., Lang A.G., Buchner A. (2007). G* Power 3: A flexible statistical power analysis program for the social, behavioral, and biomedical sciences. Behav. Res. Methods.

[40] Hosseini S.M., Shalchyan V. (2023). State-Based Decoding of Continuous Hand Movements using EEG Signals. IEEE Access.

[41] Dias E., Ulhoa P., Andrade R. Design of a 3 Degree-of-Freedom Upper-Limb Active Exoskeleton with Cable-Driven Actuators for Neuromotor Rehabilitation. Proceedings of the 2023 IEEE Colombian Caribbean Conference (C3).

[42] Dias E.A.F., Andrade R.M. (2023). Órtese Robótica de Membro Superior Movida por Cabos de Aço para Reabilitação Neuromotora. Brazil (BR).

[43] Lotte F., Bougrain L., Cichocki A., Clerc M., Congedo M., Rakotomamonjy A., Yger F. (2018). A review of classification algorithms for EEG-based brain–computer interfaces: A 10 year update. J. Neural Eng..

[44] Úbeda A., Azorín J.M., Chavarriaga R., Millán J.d.R. (2017). Classification of upper limb center-out reaching tasks by means of EEG-based continuous decoding techniques. J. Neuroeng. Rehabil..

[45] Allison B.Z., Neuper C. (2010). Could Anyone Use a BCI?. Human-Computer Interaction Series.

[46] Edelman B.J., Baxter B., He B. (2016). EEG Source Imaging Enhances the Decoding of Complex Right-Hand Motor Imagery Tasks. IEEE Trans. Biomed. Eng..

